# Supplementary material for: Actin nano-architecture of phagocytic podosomes
Source: Nat Commun. 2022 Jul 27;13:4363. doi: 10.1038/s41467-022-32038-0 (PMC9329332; doi:10.1038/s41467-022-32038-0)
Supplement: Supplementary file 1 — Supplementary Information [file 41467_2022_32038_MOESM1_ESM.pdf]

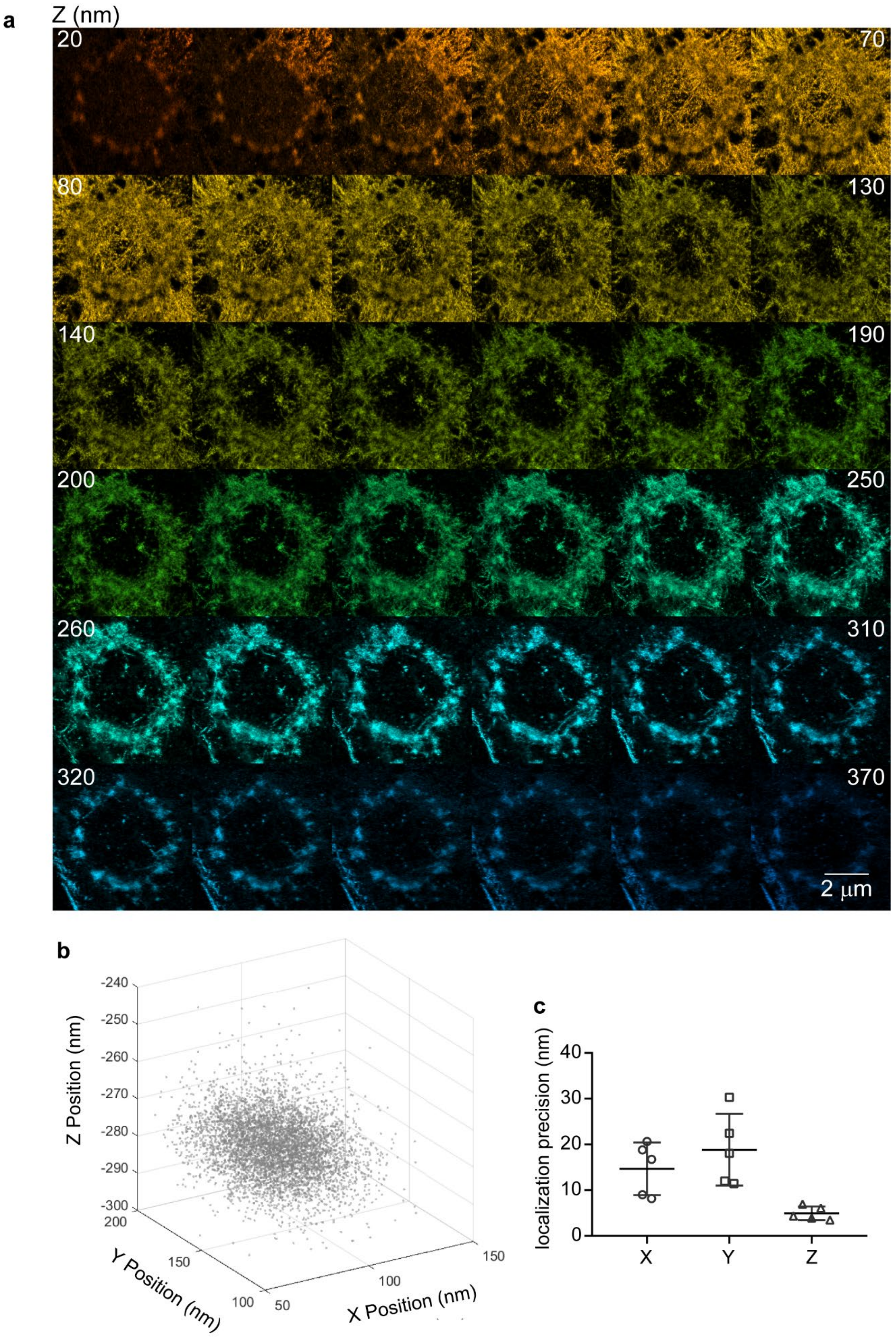
a
Z (nm)
20
70
80
130
140
190
200
250
260
310
320
370
2 μm
b
Z Position (nm)
-240
-250
-260
-270
-280
-290
-300
Y Position (nm)
200
150
100
X Position (nm)
50
100
150
c
localization precision (nm)
40
30
20
10
0
X
Y
Z

Supplementary Figure 1. Additional information for iPALM imaging. a) Additional images from the Z-stack of the frustrated phagocytosis site shown in Fig. 2a,c. Snapshots are shown at 10 nm height intervals. **b, c)** Localization precision of the iPALM system. Localization of one gold nanorod fiducial along time (randomly selected 6000 frames) is shown in **b**. Localization precision was estimated from the histogram of n = 5 nanorod positions and inferred by the standard deviation (σ = FWHM/2.3) of the distribution for each axis (shown in **c**). Whiskers show mean and one standard deviation. Source data are provided as a source data file.

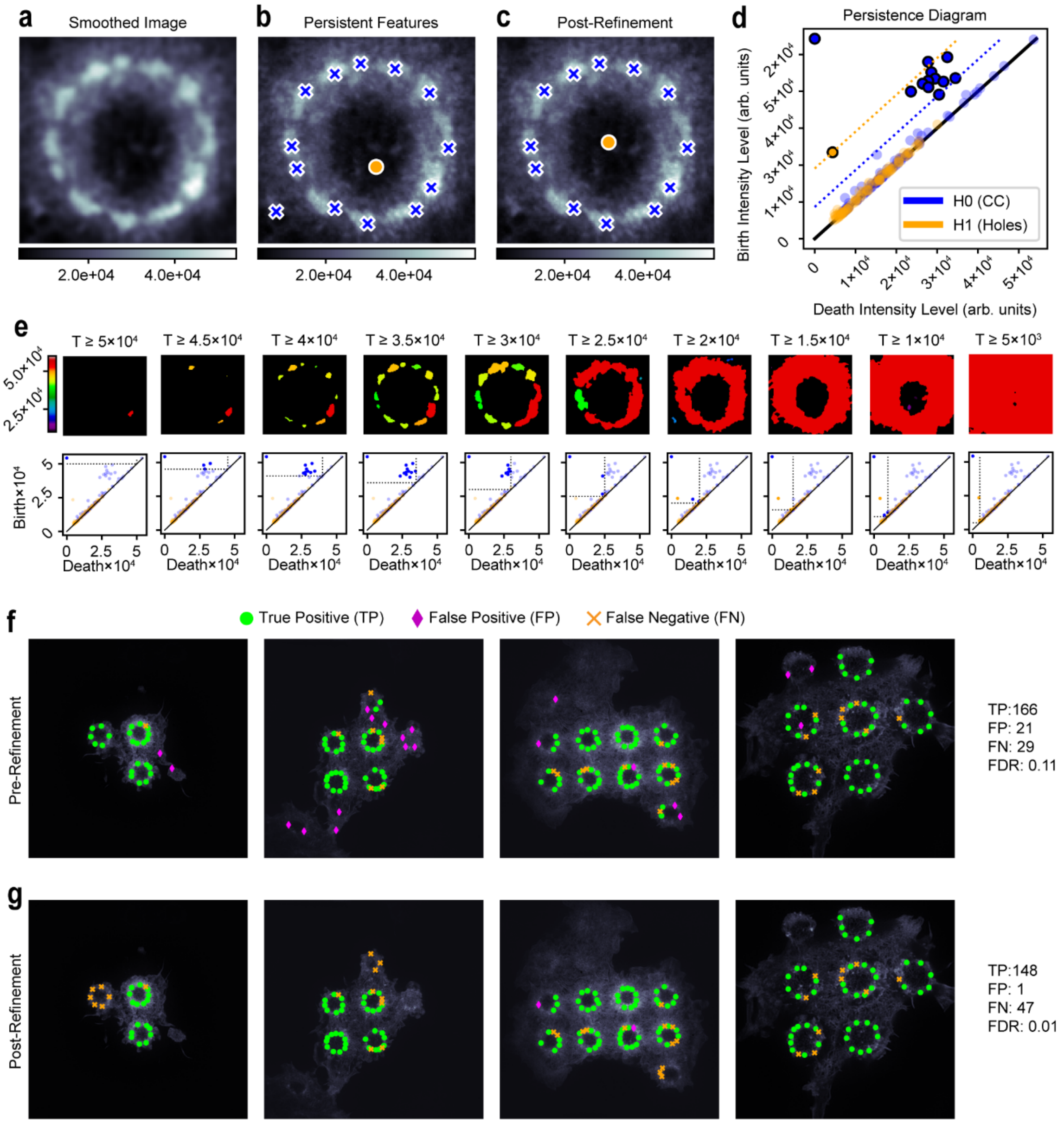

Supplementary Figure 2. Persistent homology methods for identifying podosomes and site locations. a) Smoothed image of a single phagocytosis site. An insignificant amount of noise is added for uniqueness. Color scale shows pixel intensity (arb. units). **b)** Locations of significantly persistent features from the pipeline. Color scale same as in **a**. **c)** Final locations of podosomes and the phagocytosis site centers after post-processing that excludes podosomes far away from phagocytosis sites and adjusts the locations of phagocytosis site centers. Color scale same as in **a**. **d)** Persistence diagram based on image from a. Pixels equal to the birth levels of

significantly persistent features (dots to the upper left of the persistence threshold, dotted lines) in h0 and h1 (connected components and enclosed holes respectively) are shown in b. **e)** Demonstration of level-set filtration. Visible connected components (top, colored by maximum value) and the persistence diagram (bottom, visible features correspond to top left quadrant) are shown as the level drops. **f,g)** Podosomes were manually identified if empirically determined to be associated with a well-defined phagocytosis site with at least 3 podosomes. True positives were located both manually and by the identification pipeline, false positives were located by the pipeline but not manually, and false negatives were located manually but not by the pipeline. Results are shown both before (**f**) and after (**g**) the refinement step is performed.

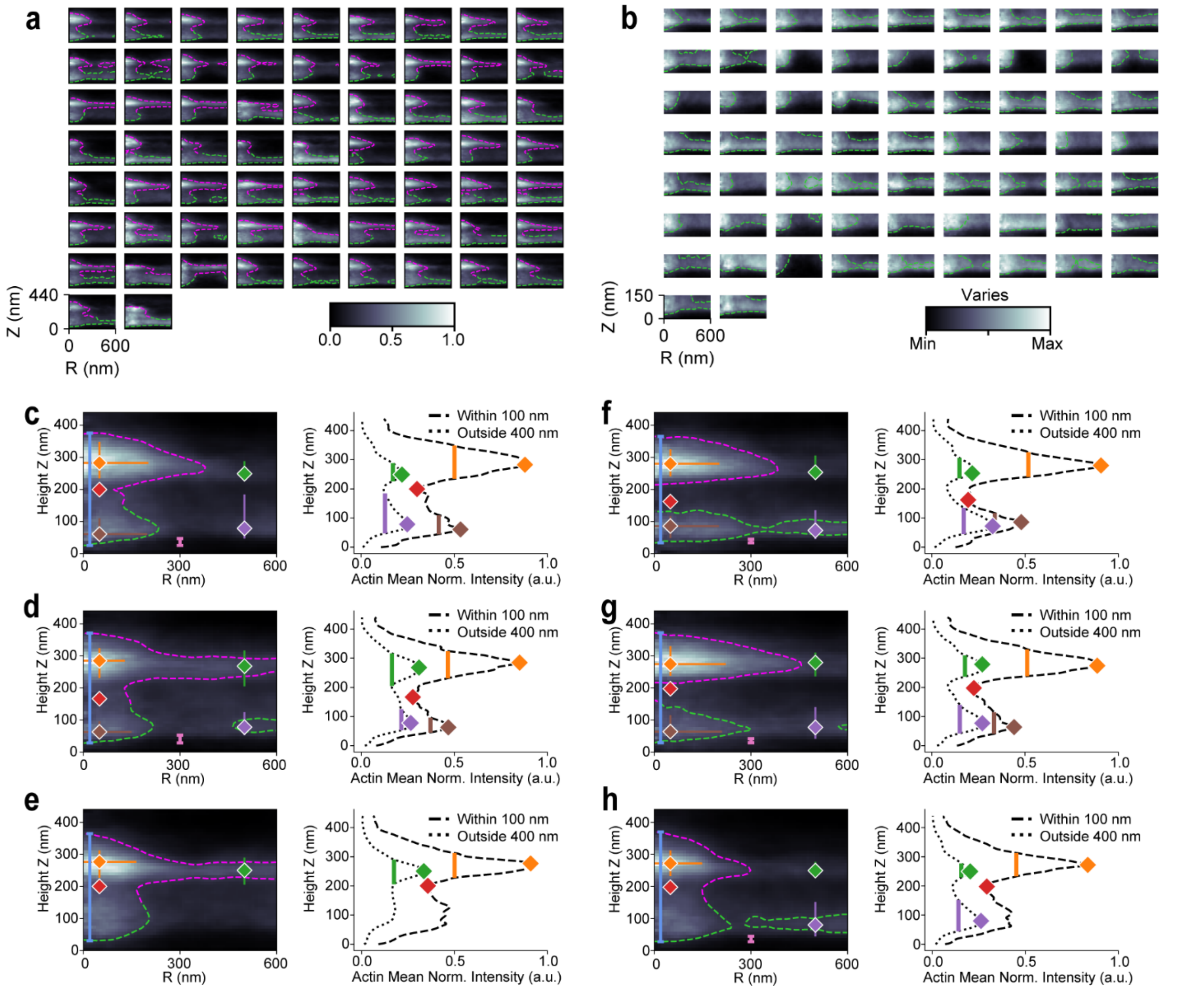

Supplementary Figure 3. Heatmaps of quantified podosomes. a) All individual radial averaging heatmaps from iPALM data, except those shown in Fig. 4f,g (n = 66 podosomes). Magenta contour based on mean actin intensity within a radius of 350 nm. Color scale shows normalized mean intensity (arb. units). **b)** All individual zoomed (Z 0 – 150 nm) radial averaging heatmaps from **a**. Each zoomed heatmap corresponds to the heatmap in the same row and column in **a**. Color scale shows normalized mean intensity (arb. units) dependent on corresponding plots in **a**. **c-h)** Additional examples of features extracted from individual radial averaging heatmaps. These examples correspond to Fig. 4d,e.

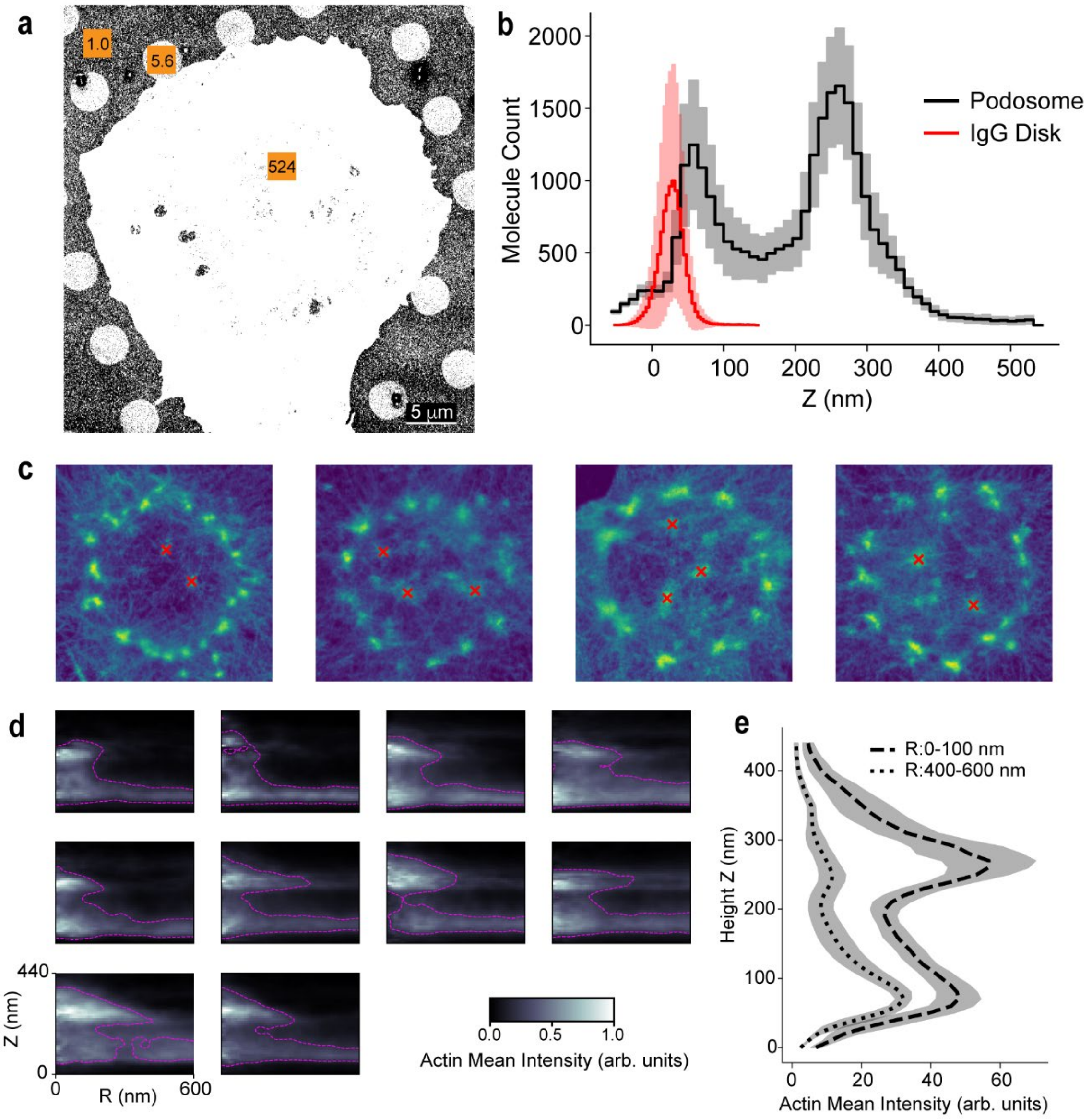

Supplementary Figure 4. Control experiments to demonstrate that IgG topology did not influence podosome morphology. a) Off-target binding of Phalloidin Alexa 647 to IgG was used for iPALM imaging of IgG disks. In the orange boxes, the normalized molecule count per μm^2 is shown for the background, within the cell, and within the IgG disks. **b)** The IgG disk (red, n = 12) is low and narrow compared to the neck and other major features of podosomes (n = 5), indicating that the bi-lobed structure of the podosome is not an artefact of formation around the edge of the IgG. **c)** iPALM imaging of podosomes that formed over IgG disks (n_{sites} = 4, n_{pod} = 10), rather than at edges. **d**) Radial averaging heatmaps of the podosomes in **c**. Magenta contour is drawn at 70% of the maximum intensity. **e)** Within 100 nm and from 400-600 nm, mean actin intensity for the heatmaps in **d**. Source data are provided as a source data file.

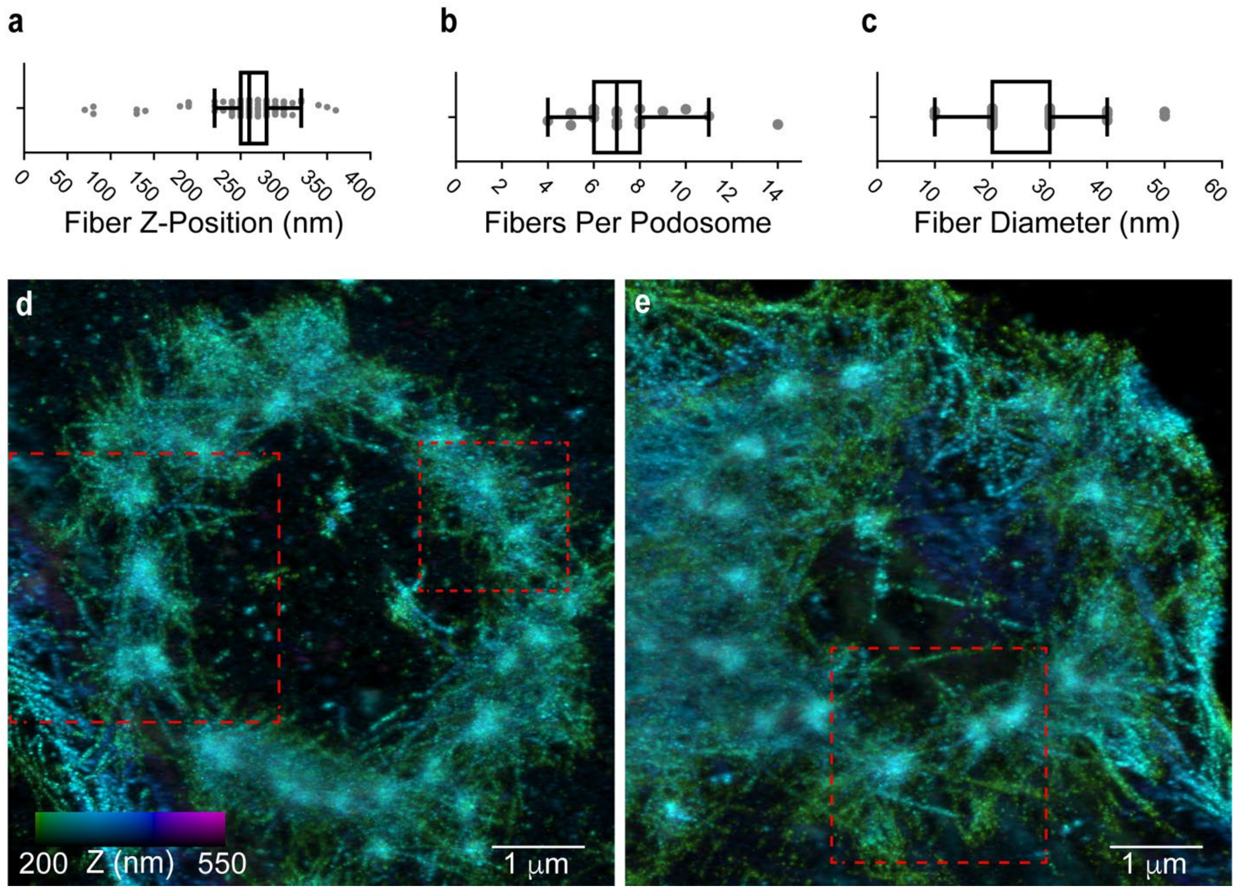

Supplementary Figure 5. Further characterization of radial filaments. a-c) Average Z-position for radial filaments, the number of filaments per podosome, and filament diameter. Measurements were extracted from Imaris analysis (n = 146 filaments across 20 podosomes). **d,e)** From two rings of podosomes, examples of color coded Z-projections. Color bar indicates z distance from 200 to 550 nm. White boxes show the images of Fig. 6e.

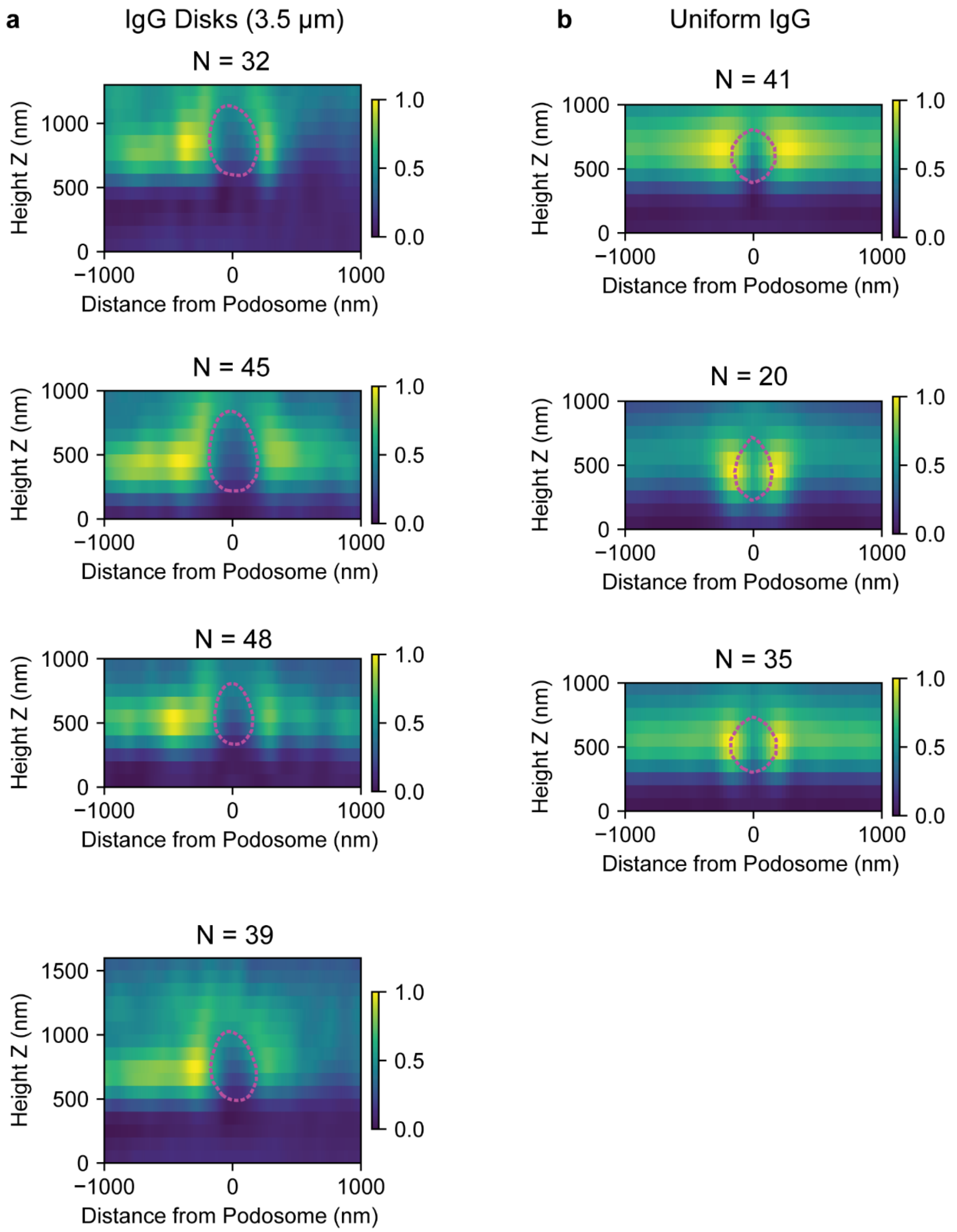

Supplementary Figure 6. 3D heatmap representations of paxillin. a,b) 3D-SIM results for paxillin on uniform antibody (**a**) or on IgG disks (**b**). On uniform IgG, the heatmaps are mirrored radial averaging heatmaps, as there is no phagocytosis site center to orient the analysis. On the IgG disks, these are the perpendicular line scan heatmaps. Each heatmap shows a single cell, and N is the number of podosomes. Magenta contour is drawn for the actin channel at 70% maximum intensity. Color scales show normalized mean intensity (arb. units).

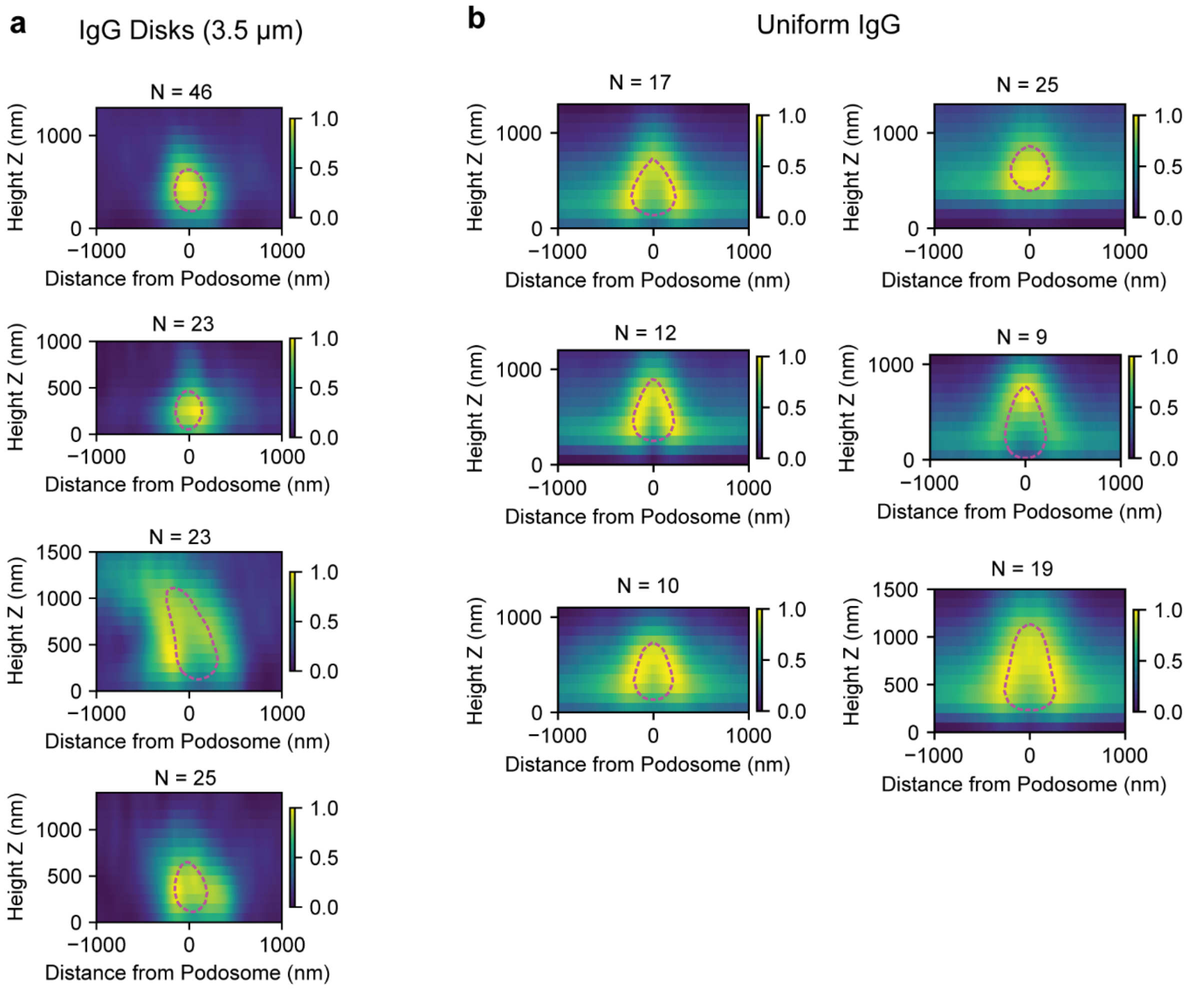

Supplementary Figure 7. Imaging of α-actinin. a,b) 3D-SIM results for α-actinin on IgG disks (**a**) or on uniform antibody (**b**). On uniform IgG, the heatmaps are mirrored radial averaging heatmaps as there is no phagocytosis site center to orient the analysis. On the IgG disks, these are the perpendicular line scan heatmaps. Each heatmap shows a single cell, and N is the number of podosomes. Magenta contour is drawn for the actin channel at 70% maximum intensity. Color scales show normalized mean intensity (arb. units).

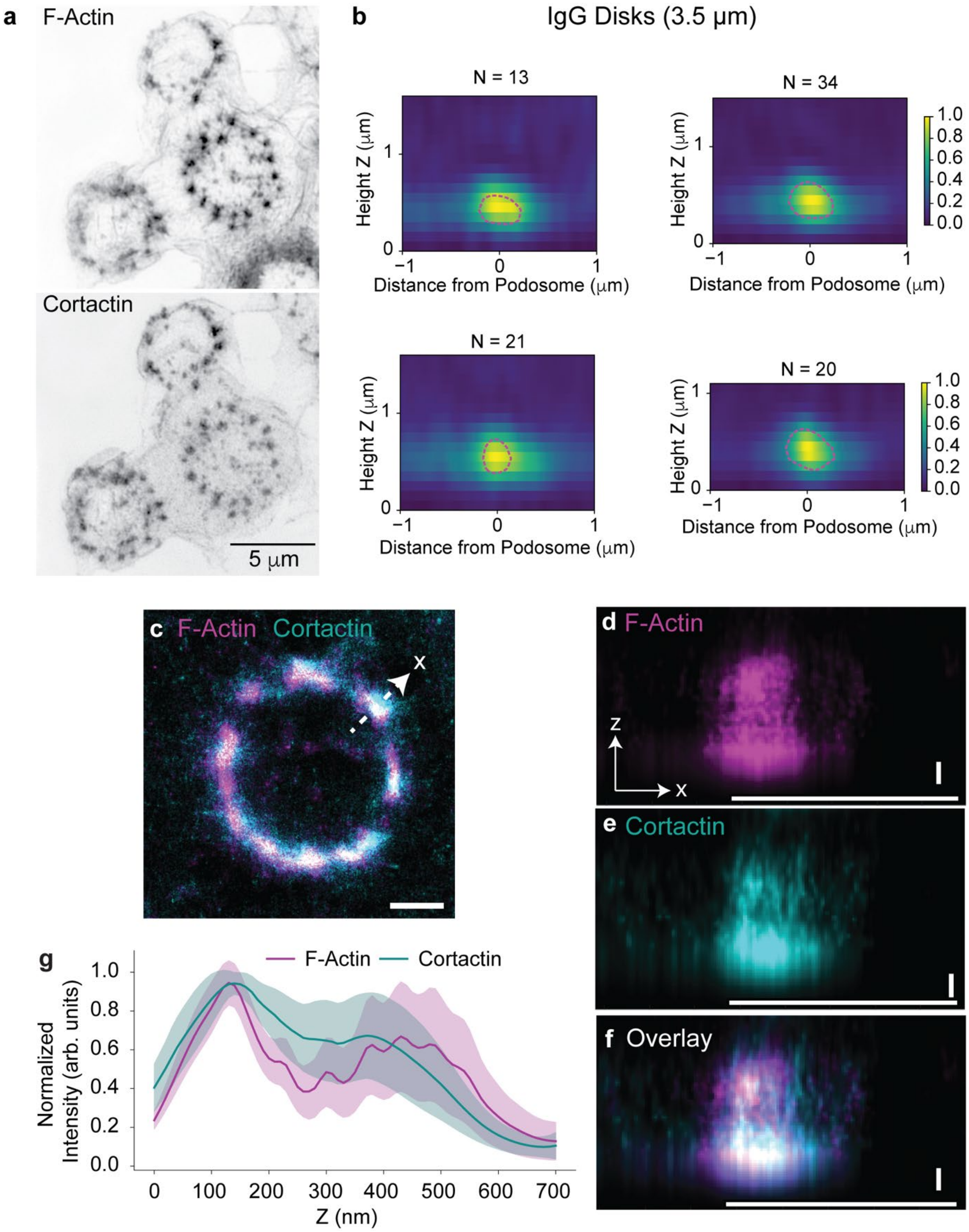

Supplementary Figure 8. Imaging of cortactin. a) Z-projections of F-actin (phalloidin Alexa Fluro 568) and EGFP-cortactin from 3D-SIM imaging. **b)** Perpendicular line scan heatmaps of cortactin from 3D-SIM. Each heatmap shows a single cell, and N is the

number of podosomes. Magenta contour is drawn for the actin channel at 70% maximum intensity. Color scales show normalized mean intensity (arb. units). **c)** Z-projection of F-actin (phalloidin Alexa Fluro 647) and mEos3.2-cortactin from 3D-PALM/STORM imaging. Scale bar is 1 micron. Representative, manually drawn line scan shown for a single podosome. **d-f)** Heatmaps for F-actin and cortactin distribution for a single podosome, from 3D-PALM/STORM imaging. X-axis scale bar is 1 micron, Z-axis scale bar is 100 nm. **g)** Normalized, mean intensity for F-actin and cortactin from n = 31 podosomes (across 4 cells), from 3D-PALM/STORM imaging. Source data are provided as a source data file.

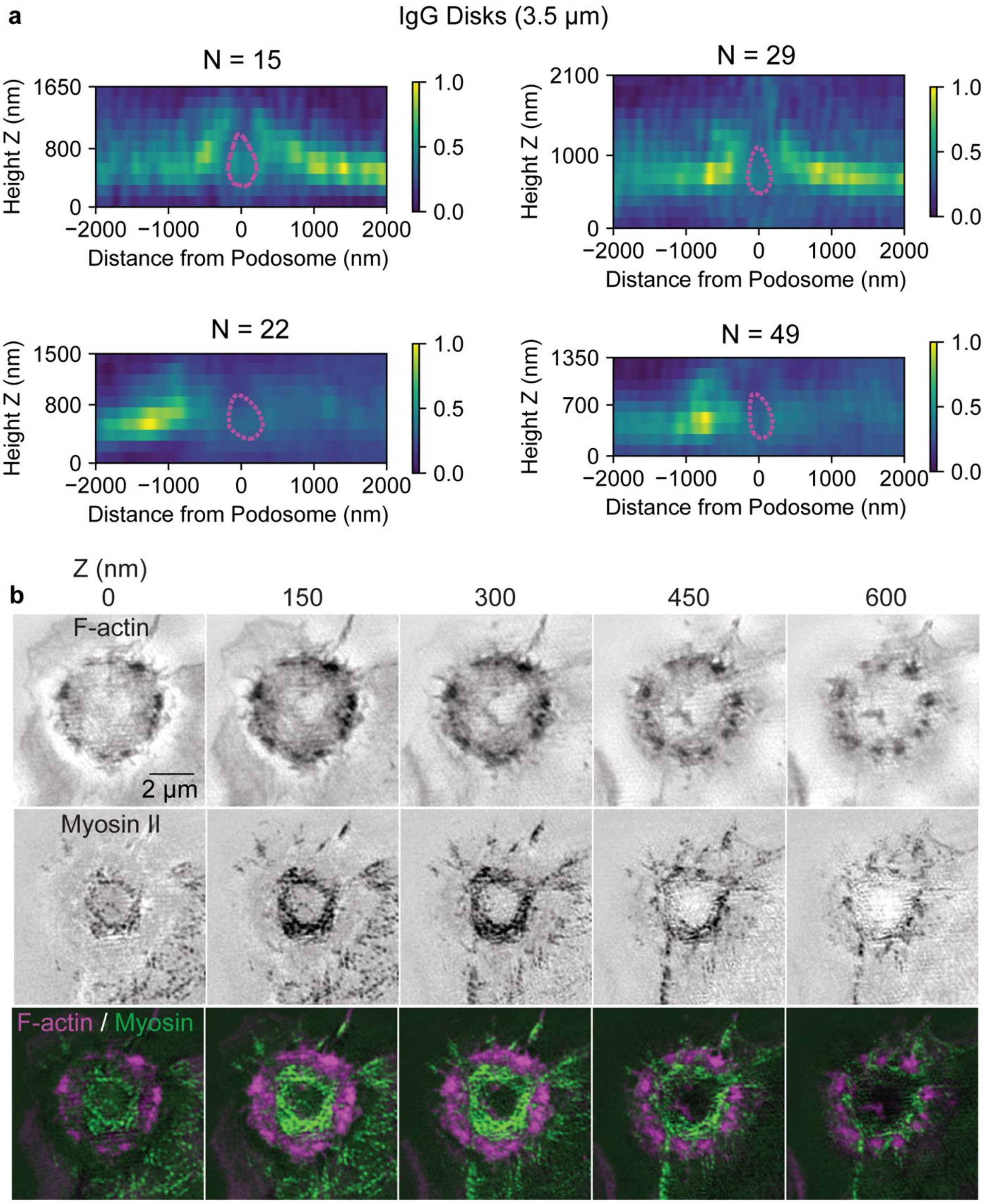

Supplementary Figure 9. 3D myosin distribution relative to podosomes. a) 3D-SIM of myosin on IgG disks (perpendicular line scan heatmaps). Each heatmap shows a single cell, and N is the number of podosomes. Magenta contour is drawn for the actin channel at 70% maximum intensity. Color scale shows normalized mean intensity (arb.

units). **b)** Additional 3D-SIM Z-stack images showing actin marked with lifeact-Halo-549, myosin II marked with RLC-EGFP, and merged.
