# Supplementary material for: Actin nano-architecture of phagocytic podosomes
Source: Nat Commun. 2022 Jul 27;13:4363. doi: 10.1038/s41467-022-32038-0 (PMC9329332; doi:10.1038/s41467-022-32038-0)
Supplement: Supplementary file 3 — Description of additional supplementary files [file 41467_2022_32038_MOESM3_ESM.pdf]

Description of Additional Supplementary Files

Supplementary Movie 1. - Primary mouse macrophage expressing FcγRIIA-EGFP (green) and LifeactmCherry (magenta) migrates over a 15 µm IgG disk, forming podosomes around the edge of the disk. The images were acquired by confocal microscopy at 5 seconds intervals.

Supplementary Movie 2. - TIRF-SIM of actin podosome ring formation during frustrated phagocytosis. RAW 264.7 macrophages expressing FTractinEGFP to mark F-actin. The duration of the movie is 4 min and 40 seconds with 5 second intervals between frames using TIRF-SIM.

Supplementary Movie 3. - iPALM Z stack of a FP site. F-actin is labeled with phalloidin Alexa 647. The colors indicate z distance of actin from 0 (red) to 550 (magenta) nm. There are 10 nm intervals between frames.

Supplementary Movie 4. - Volumetric visualization of an individual podosome. The visualization is rotated 360 degrees from the bottom to the top along the x axis. There are 2 degrees between frames. Note the actin knob extending from the bottom of the podosome.

Supplementary Movie 5. - 3D-SIM Z stack of F-actin and myosin II. RAW 264.7 macrophages expressing RLCEGFP to mark myosin II filaments (green) and lifeact-Halo-549 to mark Factin (magenta). The length of the movie is 1200 nm with 150nm intervals between frames.

Supplementary Movie 6. - Rotation of a 3D-SIM projection of F-actin and myosin II. RAW 264.7 macrophages expressing RLC-EGFP to mark myosin II filaments (green) and lifeact-Halo549 to mark F-actin (magenta). The 3D projection is rotated 90 degrees from the top to the side along x axis. There are 10 degrees between frames.

Supplementary Movie 7. - Microtubules inside a circle of podosomes. RAW 264.7 macrophages expressing a GFP fusion of the ensconsin MT-binding domain (EMTBEGFP) to mark microtubules (cyan) and Lifeact-Halo-549 to mark F-actin (magenta). The duration of this TIRFSIM movie is 1 min and 18 seconds with 2 second intervals between frames. Note the MT restricted within a circle of actin (approx. 5 o'clock).

Supplementary Movie 8. - Dynamics of microtubule plus-ends during macrophage frustrated phagocytosis. RAW 264.7 macrophages expressing EB3-EGFP to mark the plus-end of microtubules (green) and Lifeact-Halo549 to mark F-actin (magenta). The duration of this TIRF-SIM movie is 1 min and 24 seconds with 2 second intervals between frames. This movie shows MT restricted to the interior of a circle of podosomes (approx. 10 o'clock).
