# Supplementary material for: Actin nano-architecture of phagocytic podosomes
Source: Nat Commun. 2022 Jul 27;13:4363. doi: 10.1038/s41467-022-32038-0 (PMC9329332; doi:10.1038/s41467-022-32038-0)
Supplement: Supplementary file 12 — Reporting Summary [file 41467_2022_32038_MOESM12_ESM.pdf]

nature portfolio

Corresponding author(s): Klaus M. Hahn, Timothy C. Elston

Last updated by author(s): Jun 21, 2022

Reporting Summary

Nature Portfolio wishes to improve the reproducibility of the work that we publish. This form provides structure for consistency and transparency in reporting. For further information on Nature Portfolio policies, see our Editorial Policies and the Editorial Policy Checklist.

Statistics

For all statistical analyses, confirm that the following items are present in the figure legend, table legend, main text, or Methods section.

n/a	Confirmed	
☐	☒	The exact sample size (n) for each experimental group/condition, given as a discrete number and unit of measurement
☐	☒	A statement on whether measurements were taken from distinct samples or whether the same sample was measured repeatedly
☐	☒	The statistical test(s) used AND whether they are one- or two-sided<br>*Only common tests should be described solely by name; describe more complex techniques in the Methods section.*
☒	☐	A description of all covariates tested
☐	☒	A description of any assumptions or corrections, such as tests of normality and adjustment for multiple comparisons
☐	☒	A full description of the statistical parameters including central tendency (e.g. means) or other basic estimates (e.g. regression coefficient) AND variation (e.g. standard deviation) or associated estimates of uncertainty (e.g. confidence intervals)
☐	☒	For null hypothesis testing, the test statistic (e.g. F, t, r) with confidence intervals, effect sizes, degrees of freedom and P value noted<br>*Give P values as exact values whenever suitable.*
☒	☐	For Bayesian analysis, information on the choice of priors and Markov chain Monte Carlo settings
☒	☐	For hierarchical and complex designs, identification of the appropriate level for tests and full reporting of outcomes
☒	☐	Estimates of effect sizes (e.g. Cohen's d, Pearson's r), indicating how they were calculated

Our web collection on statistics for biologists contains articles on many of the points above.

Software and code

Policy information about availability of computer code

Data collection

Nikon N-SIM: Acquisition and reconstruction were carried out using the NIS-Elements software (Nikon Elements AR 4.51.01 64bit).
iPALM: Data acquisition was carried out using software written in LABVIEW (National Instruments).

Data analysis

1. IPALM image processing and rendering were performed using PeakSelector V9.5 (Janelia Research Campus, Kanchanawong et al., 2010; Shtengel et al., 2009).
2. Fiji (v2.3.0) was used to generate volumetric views ('3D Viewer'). This was also used to generate radial profiles ('Radial Profiler' plugin).
3. Imaris (v 9.5) was used to analyze and detect filaments within Z-stacks of images using 'FilamentTracer'.
4. Using Python (v 3.7.10) code and Jupyter Notebook (v 6.1.4) (as well as the packages Scipy (v 1.7.3), Dionysus (v 2.0.6)), custom code (available at https://github.com/elstonlab/PodosomeImageAnalysis, v1.1) was used to:
a. Discover podosomes and phagocytosis site locations from actin channels by applying persistent homology using the Dionysus2 package.
b. Generate 3D heatmaps showing podosomes from Z-stacks of images and perform analysis to quantify features in these heatmaps.
c. Quantify and visualize the distribution of actin with other images species from both 2D images and from Z-stacks of images.
d. Perform a 3D contour rendering of the average podosome using the Mayavi (v 4.7.2) package.

For manuscripts utilizing custom algorithms or software that are central to the research but not yet described in published literature, software must be made available to editors and reviewers. We strongly encourage code deposition in a community repository (e.g. GitHub). See the Nature Portfolio guidelines for submitting code & software for further information.

Data

Policy information about availability of data

All manuscripts must include a data availability statement. This statement should provide the following information, where applicable:

- Accession codes, unique identifiers, or web links for publicly available datasets
- A description of any restrictions on data availability
- For clinical datasets or third party data, please ensure that the statement adheres to our policy

Most of the data generated in this study have been deposited in Zenodo [https://doi.org/10.5281/zenodo.6657586]. Some of the raw image files were too large to host but these are available upon request from the corresponding authors. Source data are provided with this paper.
This statement is included in the manuscript.

Field-specific reporting

Please select the one below that is the best fit for your research. If you are not sure, read the appropriate sections before making your selection.

☒ Life sciences ☐ Behavioural & social sciences ☐ Ecological, evolutionary & environmental sciences

For a reference copy of the document with all sections, see nature.com/documents/nr-reporting-summary-flat.pdf

Life sciences study design

All studies must disclose on these points even when the disclosure is negative.

Sample size	Sample sizes were not predetermined. All podosomes with good image quality were included, provided they were associated with a well-defined ring of podosomes around an IgG circle. The number of samples is indicated in individual figure legends and in the analysis methods. Statistical analysis of each specific measurement showed that the quantitation of podosome dimensions could be carried out with a certainty of at least +/- 5%, in many cases better.
Data exclusions	Images of poor quality were excluded from analysis. Podosomes that were not associated with a well-defined ring of podosomes were excluded based on an automated process.
Replication	All the experiments were reproducible. We examined 110 podosomes from four cells, across three images. The quantitation of podosome dimensions was carried out using the 72 podosomes from the highest quality image.
Randomization	The allocation was random.
Blinding	There was no need for blinding, as data selection was based on an automated process.

Reporting for specific materials, systems and methods

We require information from authors about some types of materials, experimental systems and methods used in many studies. Here, indicate whether each material, system or method listed is relevant to your study. If you are not sure if a list item applies to your research, read the appropriate section before selecting a response.

Materials & experimental systems

n/a	Involved in the study	
☐	☒	Antibodies
☐	☒	Eukaryotic cell lines
☒	☐	Palaeontology and archaeology
☐	☒	Animals and other organisms
☒	☐	Human research participants
☒	☐	Clinical data
☒	☐	Dual use research of concern

Methods

n/a	Involved in the study	
☒	☐	ChIP-seq
☒	☐	Flow cytometry
☒	☐	MRI-based neuroimaging

Antibodies

Antibodies used	Goat anti-Mouse IgG Alexa Fluor 405 (ThermoFisher Scientific, A31553), Goat anti-Mouse IgG Alexa Fluor 647 (ThermoFisher Scientific, A21236). We also used the following reagents: Alexa Fluor 488 phalloidin (Molecular Probes A12379), Alexa Fluor 568 phalloidin (Molecular Probes A12380), Alexa Fluor 647 Phalloidin (Molecular Probes; A22287).
Validation	ThermoFisher Scientific anti-Mouse secondary antibodies are affinity-purified antibodies with well-characterized specificity for mouse immunoglobulins and are useful in the detection, sorting or purification of its specified target.

Purity of phalloidin products is 99% using the methods HPLC2. The approximate MWs, and the peak excitation and emission wavelengths are 1320 Da, 495 nm, 518 nm for Alexa Fluor 488 phalloidin; 1590 Da, 578 nm, 600 nm for Alexa Fluor 568 phalloidin; 1950 Da, 650 nm, 668 nm for Alexa Fluor 647 Phalloidin.

Eukaryotic cell lines

Policy information about cell lines

Cell line source(s)	RAW 264.7 macrophages were obtained from the ATCC (TIB-71), https://www.atcc.org.
Authentication	Authentication was done by the ATCC.
Mycoplasma contamination	The cells were routinely tested for mycoplasma contamination using a PCR detection method; results were negative.
Commonly misidentified lines (See ICLAC register)	No commonly misidentified cell lines were used in the study.

Animals and other organisms

Policy information about studies involving animals; ARRIVE guidelines recommended for reporting animal research

Laboratory animals	This study employed male and female C57BL/6 mice at the age of 8-10 weeks.<br>Mice had access to food and water ad libitum and were maintained at constant temperature (22–24 °C), humidity (40–60%), and 12 h light/dark cycles.
Wild animals	No wild animals were used in the study.
Field-collected samples	No field collected samples were used in the study.
Ethics oversight	All procedures were conducted in accordance with the NIH Guide for the Care and Use of Laboratory Animals and with the approval of the Institutional Animal Care and Use Committee at the University of North Carolina at Chapel Hill.

Note that full information on the approval of the study protocol must also be provided in the manuscript.
